# Emotional speech markers of psychiatric disturbance in Huntington’s disease

**DOI:** 10.3389/fpsyt.2025.1633492

**Published:** 2025-08-12

**Authors:** Lucie Chenain, Audrey Fabre, Hadrien Titeux, Graça Morgado, Katia Youssov, Chloé Clavel, Anne-Catherine Bachoud-Lévi

**Affiliations:** ^1^ NeuroPsychologie Interventionnelle, Département d’Etudes Cognitives, Ecole Normale Supérieure, Paris Sciences et Lettres University, Paris, France; ^2^ NeuroPsychologie Interventionnelle, Université Paris Est Créteil, Institut national de la santé et de la recherche médicale (INSERM) Unité 955, Institut Mondor de Recherche Biomédicale, Créteil, France; ^3^ Automatic Language Modelling and Analysis & Computational Humanities (ALMAnaCH), Institut national de recherche en sciences et technologies du numérique (INRIA), Paris, France; ^4^ NeurATRIS, Hôpital Henri Mondor, Créteil, France; ^5^ Learning Planet Institute, Université Paris Cité, Paris, France; ^6^ Assistance Publique — Hôpitaux de Paris (AP-HP), Hôpital Henri Mondor-Albert Chenevier, Centre de Référence Maladie de Huntington, Service de Neurologie, Créteil, France; ^7^ Inserm, Centre d’Investigation Clinique 1430, AP-HP, Hôpital Henri Mondor, Créteil, France; ^8^ Laboratoire de Sciences Cognitives et Psycholinguistique, Centre national de la recherche scientifique (CNRS), Unité 8554, Paris Sciences et Lettres (PSL) University, Paris, France

**Keywords:** Huntington’s disease, emotional expression, psychiatric symptoms, speech analysis, remote monitoring

## Abstract

**Introduction:**

Psychiatric disorders and difficulties in emotional expression represent a major problem in the management of Huntington’s Disease (HD). To improve patient follow-up, we propose to investigate the link between emotional expression and psychiatric symptoms, measured by the Problem Behaviors Assessment (PBA) scale. To this aim we developed the first emotional/psychiatric speech corpus, emoHD.

**Methods:**

We included 102 HD gene carriers and 35 healthy controls (HC). Psychiatric symptoms were assessed using PBA sub-scales for Depression, Irritability/aggressivity, Apathy, and Obsessive/compulsive symptoms. Speech was annotated using three emotional descriptors: primary emotions, affective phenomena, and activation levels. Affective phenomena labels were selected based on PBA statements by external participants unaware of the study’s aims. We analyzed (1) emotional descriptors’ relationships, (2) emotional expression differences between HD and HC, and (3) the associations between emotions and psychiatric symptoms.

**Results:**

HD patients showed reduced emotional expressiveness than HC with more neutral activation levels (=0). Only the primary emotion “angry” was less expressed in HD compared to HC. In contrast they expressed more affective phenomena states like apathetic, confused, “depressed”, “disoriented”, “frustrated”, and “pessimistic” than HC, whereas they expressed less “other” and “irritable” than HC. Expressed emotions were congruent with psychiatric symptoms (e.g., “anxious” and “nervous” are positively associated with Depression PBA sub-scale; “frustrated” with Irritability/aggressivity PBA sub-scale).

**Conclusion:**

We showed that speech is a promising marker for emotional/psychiatric symptoms in HD, supporting future remote monitoring and personalized care strategies.

## 1 Introduction

Huntington’s Disease (HD) is an autosomal dominant neurodegenerative disorder caused by the repeated expansion of CAG trinucleotides in the huntingtin gene, with full penetrance achieved with ≧ 39 repetitions (1). It is characterized by motor, cognitive and psychiatric symptoms, leading to disability and gene carriers’ death close to 20 years after disease onset (2). Its large spectrum of symptoms makes it a valuable model for studying other neurodegenerative disorders.

Indeed, HD individuals experience both psychiatric disorders and emotional production and perception deficits for facial, body, and vocal expressions (3–7). On one hand, ineffective emotional processing can impact interpersonal relations and contribute to emotional reasoning difficulties, which may underlie or interact with patients’ psychiatric profiles (8). On the other, HD individuals exhibit risky behaviors: they have the highest rate of suicide among those with a diagnosed neurological disorder, with an absolute risk of 1.6 (9). Irritability is common, affecting 38-73% of individuals, and may lead to hetero-aggressive and auto-aggressive behaviors. Other psychiatric behaviors include depression (prevalence of 20-56%), anxiety (16.7-24%), apathy (34-76%), and psychosis (10.4%) (8). These symptoms affect patients’ daily functioning (10–12), quality of life (13–15), and relationships, often causing social withdrawal, and family breakups (16–18). While pharmaceutical treatments and psychological support may relieve symptoms (19), their detection and monitoring remains a challenge, presumably, because of patients’ difficulties in expressing emotion. Yet, the absence of link between emotions and psychiatric behaviors represents a limitation.

This highlights the need to investigate the interplay between emotional deficits and psychiatric manifestations in HD. Several factors make speech a valuable medium for gathering such information. Speech carries a communicative message through linguistic content along with paralinguistic cues, which are defined as nonverbal forms of communication that contribute to conveying the message’s meaning. Its production is intrinsically influenced by a speaker’s physical and psychological status changes, altering their vocal apparatus control, even subconsciously (20). Speech recordings of individuals with self-reported or clinically diagnosed psychiatric disorders have been used to analyze psychiatric symptoms. This has contributed to the development of speech corpora with non-dysarthric patients, such as the AVEC, RAPID and DEPAC corpora for depression (21–23), MONARCA for bipolar disorder (24), Etude 1000 (25) for post-traumatic stress disorder, and the BBRS (26) or Scherer et al. (27) for suicide. Such databases can identify psychiatric behaviors (28, 29), support differential diagnosis (30, 31) and predict the onset of a psychiatric disorder in at-risk individuals (31). Speech also carries acoustic cues of emotions, such as prosody, intensity, and speech rate (32), motivating the development of emotional speech corpora where recordings are annotated with emotional descriptors to capture various emotional information (33). In neurological populations, such corpora are used for emotional analysis (34) and the development of speech emotion recognition models (35, 36). However, the underrepresentation of pathological speakers and the lack of psychiatric information collected (37–41) can make generalization and real-world applications difficult. The development of disorder-specific emotional speech corpora that include clinical and psychiatric assessment of speakers could therefore improve the generalizability and applicability of findings.

**Figure 1:**
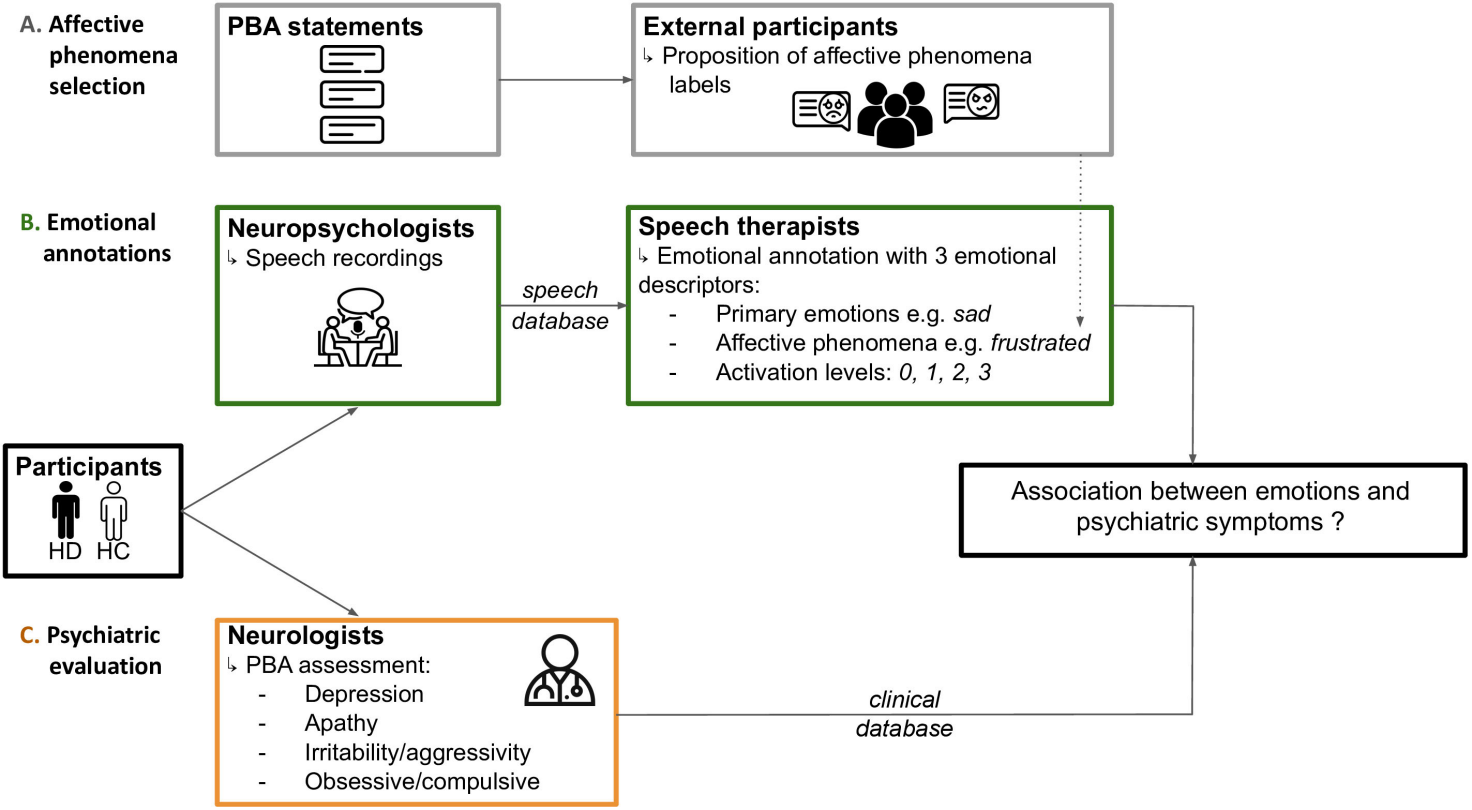
Overview of the emoHD corpus. Participants in the emoHD corpus were Huntington’s Disease (HD) gene carriers’ and healthy controls (HC). They underwent psychiatric evaluation (pathway C) by certified neurologists (orange box) using the Problem Behaviors Assessment (PBA) sub-scales, constituting the clinical database of emoHD. Independently, participants’ speech was recorded by neuropsychologists (green box), constituting the speech database of emoHD. Recordings were emotionally annotated (pathway B) by speech therapists (green box) which included three emotional descriptors: (1) primary emotions for coarse-grain characterization; affective phenomena to capture emotional manifestations specific to Huntington’s disease — these labels were selected by external participants (grey box) to maintain all steps independent and blind to each other (pathway A); and (3) activation levels to indicate the emotional excitation expressed. Researcher (LC) was blind to the participants’ status, and ran the statistical analysis.

We developed the HD emotional speech corpus (emoHD) using recordings from participants psychiatrically evaluated by certified neurologists, with emotional annotations provided by speech therapists blinded to the neurological assessment. Emotions were annotated using three emotional descriptors: first, primary emotions were annotated, referring to universal, cross-cultural categories such as Ekman’s six (42), providing a basic emotional characterization. Second, affective phenomena, as defined by Scherer (43), which include a broader range of emotional and affective states including: feelings referring to subjective experience of emotion; moods which are diffuse and longer lasting affective states; preferences which are stable evaluative judgments; attitudes for long-lasting predispositions or beliefs; affective dispositions which are personality traits predisposing individuals to certain emotions or moods; and interpersonal stances which are affective styles that develop during interactions with others. To select a list of affective phenomena labels, external participants reviewed statements extracted from the standardized HD psychiatric scale (the Problem Behaviors Assessment - short form (PBA) (44)) and identified the emotional manifestations they perceived in each. These responses informed the selection of labels used to annotate patients’ speech. These external participants were blind to the study’s objectives, unfamiliar with HD, and did not know the clinical significance of the PBA. Finally, speech therapists rated the activation levels, representing the emotional excitation expressed through physiological changes (heart rate increase, internal temperature rising, etc.) (45) (**Figure 1**).

With emoHD, we (1.) investigated and validated the annotation scheme by establishing how the different emotional descriptors are associated with each other; (2.) explored how emotional expression differs between HD gene carriers and controls; and (3.) assessed how emotional expression relates to psychiatric profiles. This allowed us to propose a framework for the use of emotional speech analysis as a potential marker for psychiatric symptoms in HD to constitute the first step towards patient remote monitoring of emotional and affective disturbances based on speech analysis. It will likely contribute to targeted interventions and personalize care strategies for individuals living with HD and may ultimately be transferred to other neurodegenerative diseases.

## 2 Materials and methods

### 2.1 Data collection

#### 2.1.1 Participants

The emoHD cohort collected clinical information and recordings from 137 participants from two observational cohorts REPAIR-HD and BIO-HD (NCT03119246 & NCT01412125) at the Henri Mondor Hospital (Créteil, France), with optional longitudinal follow-up. To constitute the EmoHD cohort, we included only the first visits of subsequent participants who had performed on the same day both speech recordings and a general clinical evaluation, using the Unified Huntington’s Disease Rating Scale (UHDRS) (46), the international reference scale in HD. All participants were French native speakers, aged ≥ 18 years, and signed an informative consent form. Exclusion criteria were incapacity to consent and significant neurological or psychiatric comorbidities unrelated to HD. Ethics approval was given by the institutional review board from Henri Mondor Hospital (Créteil, France) for the BIO-HD cohort and the CPP Saint Louis French part of the REPAIR-HD cohort, in compliance with the Helsinki Declaration.

Participants consisted of 102 HD gene carriers at various stages with a CAG repeat expansion ≥ 40 and 35 healthy controls (HC) without any cognitive deficits [Mattis Dementia Rating Scale ≥ 136 (47)]. HD gene carriers were classified on the Integrated Staging System (HD ISS): 23 gene carriers were classified in stages 0–1 corresponding to no symptoms or biomarker of cerebral volume pathogenesis, 25 in stage 2 showing symptoms of HD and 54 in stage 3 with functional change associated to HD (respectively HD ISS 0-1, 2, and 3) (48).

In addition, 96 external participants were recruited to answer an online Google form to establish a list of affective phenomena related to HD (see 2.2). They were French-speaking participants unfamiliar with HD and the PBA, blind to the study’s objective, and not working in a health-related field (see **Supplementary Table 1**).

#### 2.1.2 General clinical evaluation

Certified neurologists evaluated motor symptoms with the UHDRS Total Motor Score (TMS) and daily life functioning with the Total Functional Capacity (TFC) (46, 49). Cognitive evaluation was performed with the UHDRS verbal fluency (under 1 minute), the Stroop word, color, interference test, and the symbol digit modalities test (SDMT) (46).

Psychiatric evaluation was conducted using the PBA (44), a semi-structured diagnostic questionnaire designed to assess eleven behavioral items specific to HD: depressive mood, suicidal ideation, anxiety, irritability, angry or aggressive behavior, apathy, perseverative thinking or behavior, obsessive-compulsive behavior, delusions, hallucinations, and disoriented behavior. Neurologists rated the frequency of each behavioral item (0 = never/almost never, 1= seldom (less than 1/week), 2= sometimes (up to 4/week), 3 = frequently (most days or 6 to 7/week), 4 = daily or almost daily) and its severity (0 = absent, 1 = slight, questionable, 2 = mile (present, not a problem), 3 = moderate (symptom causing problem), 4 = severe (almost intolerable for carer).

The behavioral items scores are combined to create five PBA sub-scale scores, which are calculated as the sum of the product of each item’s severity and frequency (50, 51): the Depression sub-scale combining depressive mood, suicidal ideation and anxiety; the Irritability/aggressivity sub-scale gathering irritability and angry or aggressive behavior items, the Apathy sub-scale with the apathy item only; and lastly the Obsessive/compulsive sub-scale with perseverative thinking or behavior and obsessive-compulsive behaviors. We excluded the psychosis sub-scale, with delusions and hallucinations, as only one participant scored ≧ 1.

#### 2.1.3 Speech recordings

The speech recordings were collected as part of the BasalVoice speech recording protocol (52, 53), which included 8 speech tasks (approximately 10 minutes) among which 5 were retained for the emoHD corpus. The order of the tasks altered elicited emotional and non-emotional speech to avoid emotional contamination between tasks: counting from 1–20 forward/backwards, narrating one’s last 24 hours, then a sad story, reciting the twelve months in a year forward/backwards with hands in front and eyes closed, narrating the little riding hood tale, an anger story, describing the cookie theft picture (CTP) (54), and finally narrating a happy story to avoid ending the interview with a negative emotion. Three tasks were not considered in this study: the little riding hood task whose assessment varied among participants depending on whether they used a picture for assistance, and the forward/backward counting and month recitation which were designed to increase cognitive load by requiring participants to lift their hands and close their eyes.

Emotional speech tasks consisted of narrative prompts based on Ekman’s set of basic emotions. The narration of emotions is an effective way for speakers to express, understand and share them. The successful completion of that task favors the elicitation of emotions in speakers (55, 56). In other terms, if someone really aims to tell a sad story, we expect that they should express markers of sadness at some points. Instructions were for the sad story: “*Can you tell me a story that you find sad, really unhappy, or really depressing?*”; the anger story: “*Can you tell me a story that you find irritating, upsetting, a really annoying story?*; and the happy story: “*Can you tell me a pleasant story, full of happiness and joy, something uplifting?*”. Relevance of eliciting fear, disgust, and surprise did not appear relevant regarding the behavioral evaluation.

Non-emotional speech tasks included eliciting speech without emotional prompting by having individuals narrate their last 24 hours (“*Can you tell me what you’ve been doing for the last 24 hours?*”). This recorded natural expressions of neutral or emotional speech, depending on their recent personal experience. The CTP from the Boston Diagnostic Aphasia Examination traditionally assesses language abilities in aphasia (54) and is widely used as a speech evaluation in neurodegenerative diseases (57). Participants were shown the CTP picture and instructed “*Could you please describe everything you see in the picture?”.*

All participants were recorded under the same conditions in a hospital consultation room just after their neuropsychological assessment. They sat at a table facing the neuropsychologist, with the recording device placed 20 cm from the participant. The device used was a ZOOM H4n Pro recorder, with a sampling rate of 44.1 kHz and a 16-bit resolution. The neuropsychologists could easily set up the recording device without the need of an acoustic engineer. Participants could stop the session anytime. The duration of each speech task in recordings was extracted with python *SpontaneousCHAT* package.

### 2.2 Annotation protocol

A preliminary transcription of the linguistic content was performed by speech therapists using Praat (58) following the BasalVoice protocol (52, 53). The emoHD annotation campaign was managed on Seshat (59), a tool to automate the management of annotation campaigns of audio and speech data, by managing file handling, non-conformity and annotation inconsistency. Three speech therapists, blinded to the study’s aim, listened to the recordings and divided them in emotional segments on Praat, defined as sections of speech with a homologous emotional content (60). They were asked to label each segment with the three emotional descriptors:

- First, during the initial listening and segmentation, the speech therapists labeled each segment in one of the emotions intended to be elicited by the emotional speech tasks, namely *triste (sad)*, *joie (happy), colère (angry)*, adding *neutre (neutral)* to infer a referential neutral emotional state and *autre (other)* for all other emotions.
- Then, during a second listening of the recordings, the speech therapists annotated the same emotional segments with one or more labels corresponding to affective phenomena. The list of affective phenomena labels was defined by external participants who were blind to the study. They provided the word describing the emotional manifestations for each of the 33 PBA statements, which corresponded to the severity scale, across the 11 PBA items, ranging from mild (=2) to severe (=4). (as described in 2.1.2). The order of appearance of the statements was randomized so that the participants remained blind to the item and its severity. After converting all answers in their adjective form, the affective phenomena labels with a frequency ≧ 10% per statement were kept. This yielded a list of 23 affective phenomena labels related to HD behavior, which were used for annotation: *triste (sad), déprimé.e (depressed), désespéré.e (hopeless), pessimiste (pessimistic), détresse (distressed), anxieux.se (anxious), inquiet (worried), stressé.e (stressed), angoissé.e (nervous), peureux.se (fearful), irritable (irritable), coléreux.se (quick tempered), frustré.e (frustrated), rageant.e (infuriating), violent.e (violent), apathique (apathetic), obsessionnel.le (obsessive), délirant (delusional), désorienté.e (disoriented), perdu.e (lost)*, and *confus.e (confused).* We also added the affective phenomena *autre (other affective phenomena)* and *neutre (neutral affective phenomena)* as complementary.
- Last, activation levels of each segment were scored from 0 (no activation, equivalent to neutral) to 3 for high activation, representing the emotional excitation expressed through physiological changes (45).

The speech therapists could also add comments to the emotional segments which could be other emotions or observations.

This approach resulted in an unbiased selection of labels with intelligible common vocabulary. After annotating the whole corpus, the duration (in seconds) and the count of occurrence of each primary emotion, affective phenomenon and activation level were measured for each participant. To account for variations in recording length between individuals, the proportion of each emotional label was calculated by dividing the total duration of each label in the recording by the overall recording time.

The annotation protocol was pretested on 10 randomly selected recordings from participants’ follow-up visits (which were not included in the emoHD corpus). Agreement between the three speech therapists was obtained with the γ-agreement (61) using its *pygamma* python implementation (62) (**Table 1**). The γ-gamma is an agreement measure evaluating both the recording’s segmentation into emotional segments and their labeling, allowing for penalties in cases of discrepancies between annotators. In contrast to Cohen’s kappa or Krippendorff alpha, the γ-gamma has not been benchmarked yet, hence no threshold measure of agreement has been defined. Considering that the γ-gamma is more conservative than Cohen’s kappa or Krippendorff’s alpha, obtaining γ-gamma measures all equal or greater than 0.76 considered good.

**Table 1:** γ-gamma inter-rater agreement.

Emotional descriptor	Mean	SD	Min	Max
Average	0.76	0.11	0.56	0.91
Primary emotions	0.81	0.10	0.65	0.93
Affective phenomena	0.76	0.10	0.56	0.92
Activation	0.76	0.11	0.55	0.90

The average γ-gamma agreement is the average γ-gamma score across primary, affective phenomena and activation annotations. Standard deviations (SD), minimum range (min), maximum range (max).

### 2.3 Statistical analysis

We employed non-parametric statistical approaches to perform categorical associations, adapted to the sample size and characteristics of our population sample. This enhanced the statistical power of our analysis and accounted for the lack of distributional assumptions. We applied permutation testing to investigate the associations between emotional descriptors and emotional expression differences across HD clinical stages (63, 64).

#### 2.3.1 Associations between emotional descriptors

We examined the correspondence between the labelling of emotional descriptors (primary emotions, affective phenomena and activation levels) in each emotional segment. We first conducted a two-sided Fisher’s exact test with simulated p-values with 10000 replications. We then ran a pairwise Fisher’s exact test to evaluate the individual associations between each pair of labels: primary emotion vs affective phenomena, affective phenomena vs activation and primary emotion vs activation. Each pairwise test was corrected for multiple comparisons, adjusting the p-values using Bonferroni correction to account for possible type I errors. Odds-Ratio (OR) of each comparison was estimated to facilitate the interpretation (65, 66).

#### 2.3.2 Emotional expression in Huntington’s disease gene carriers and healthy controls

We explored the difference in emotional expressions between HD gene carriers and HC by conducting a 10000 permutation-based t-test on the proportions of emotional labels (primary emotions, affective phenomena, and activation levels). This approach randomly reshuffles group labels to generate a null distribution of t-statistics, allowing for the assessment of group differences without assuming normality. For the emotional labels with significant proportions differences (permutation p-values ≦ 0.05), we further proceeded with a 10000 permutation ANOVA comparing the HD ISS stages (HD ISS 0-1, 2 and 3) and controls, followed by pairwise comparison using Tukey’s test. This identified differences between specific clinical group status.

#### 2.3.3 Association between emotional expression and psychiatric behaviors

We explored the association between emotional expression in the emoHD corpus and psychiatric symptoms with a multilinear regression analysis. The regressions were performed to test the associations between the proportions of primary emotions, affective phenomena and activation levels with the participants’ psychiatric sub-scale scores (Depression, Irritability/aggressivity, Apathy and Obsessive/compulsive).

For each psychiatric sub-scale, a multilinear regression used individuals’ proportions as predictors, while adjusting for sex, age and the clinical group status (HD ISS 0-1, 2, 3 and HC). A bidirectional stepwise selection method was applied to identify the best predictors explaining the variance (R2) in the sub-scale scores. The statistical significance of each predictor was defined with a p-value ≦ 0.05.

All statistical analysis were performed in R (version 4.4.2), using the *stats, dplyr, multcomp, lmPerm, coin and ggplot2* and *sjPlot* packages. All preprocessing and statistical analysis are available on https://gitlab.cognitive-ml.fr/lchenain/emohd_corpus.git and are suited for python and R integrated development environments.

## 3 Results

### 3.1 Clinical assessment

The demographic and clinical information of the participants are summarized in **Table 2**. An ANOVA revealed significant differences in sex and age, the last explained by HD carriers in stages 0–1 being younger than more advanced HD stages and controls. Higher TMS and lower TFC scores, indicating worse motor and functional performance in HD carriers at stages 2 and 3, resulting in overall group differences. Similarly, cognitive evaluation indicated worse performances of HD carriers at stages 2 and 3 compared to HC and HD carriers in stage 0-1.

**Table 2:** Demographics.

Group	HC (N=35)	HD (N=102)			Group comparison & post-hoc
HD ISS		0-1 (N=23)	2 (N=25)	3 (N=54)	
***Age***					***0.033***
*Mean (SD)*	*53.937 (8.263)*	*47.338 (6.652)*	*53.445 (12.541)*	*54.226 (10.202)*	*ISS 3 > ISS 0-1*
*Range*	*36.701 - 71.140*	*26.793 - 55.918*	*25.391 - 73.029*	*32.249 - 74.105*	
***Sex***					***0.016***
*Female*	*15 (42.9%)*	*15 (65.2%)*	*7 (28.0%)*	*33 (61.1%)*	*ISS 3 > ISS 2* *ISS 3 > ISS 0-1*
*Male*	*20 (57.1%)*	*8 (34.8%)*	*18 (72.0%)*	*21 (38.9%)*	
***CAG***					
*Mean (SD)*		*41.609 (1.530)*	*42.720 (2.227)*	*44.019 (3.183)*	
*Range*		*40.000 - 45.000*	*40.000 - 49.000*	*40.000 - 55.000*	
***TMS***					***< 0.001***
*Mean (SD)*	*0.529 (1.022)*	*0.174 (0.491)*	*19.955 (13.418)*	*36.148 (13.564)*	*ISS 3 > ISS 2 > ISS 0–1 ISS 3 > ISS 2 > HC*
*Range*	*0.000 - 5.000*	*0.000 - 2.000*	*0.000 - 42.000*	*1.000 - 60.000*	
***TFC***					***< 0.001***
*Mean (SD)*	*13.000 (0.000)*	*12.913 (0.417)*	*12.636 (0.581)*	*9.667 (2.037)*	*ISS 3 > ISS 2 > ISS 0-1* *ISS 3 > ISS 2 > HC*
*Range*	*13.000 - 13.000*	*11.000 - 13.000*	*11.000 - 13.000*	*4.000 - 12.000*	
***Verbal fluency***					***< 0.001***
*Mean (SD)*	*42.237 (11.322)*	*45.957 (10.218)*	*28.360 (13.038)*	*21.056 (9.303)*	*HC > ISS 2 > ISS 3* *ISS 0-1 > ISS 2 > ISS 3*
*Range*	*17.000 - 66.000*	*25.000 - 72.000*	*10.000 - 57.000*	*3.000 - 50.000*	
***SDMT***					***< 0.001***
*Mean (SD)*	*50.289 (7.580)*	*55.565 (8.938)*	*32.480 (12.039)*	*23.704 (9.123)*	*HC > ISS 2 > ISS 3* *ISS 0-1 > ISS 2 > ISS 3*
*Range*	*34.000 - 69.000*	*41.000 - 78.000*	*17.000 - 61.000*	*3.000 - 45.000*	
***Stroop word***					***< 0.001***
*Mean (SD)*	*104.211 (12.003)*	*102.130 (11.659)*	*76.320 (19.304)*	*57.434 (15.859)*	*HC > ISS 2 > ISS 3* *ISS 0-1 > ISS 2 > ISS 3*
*Range*	*84.000 - 137.000*	*80.000 - 122.000*	*47.000 - 114.000*	*23.000 - 97.000*	
***Stroop color***					***< 0.001***
*Mean (SD)*	*79.368 (9.301)*	*77.739 (12.050)*	*56.840 (15.418)*	*43.750 (13.435)*	*HC > ISS 2 > ISS 3* *ISS 0-1 > ISS 2 > ISS 3*
*Range*	*56.000 - 93.000*	*48.000 - 107.000*	*28.000 - 86.000*	*16.000 - 85.000*	
***Stroop interference***					***< 0.001***
*Mean (SD)*	*46.342 (8.325)*	*47.261 (11.189)*	*32.560 (11.329)*	*23.231 (7.432)*	*HC > ISS 2 > ISS 3* *ISS 0-1 > ISS 2 > ISS*
*Range*	*30.000 - 65.000*	*27.000 - 70.000*	*11.000 - 53.000*	*7.000 - 38.000*	

Demographic and clinical assessments of healthy controls (HC) and Huntington’s disease (HD) gene carriers grouped according to the Integrating Staging System (ISS). All scores are displayed with the mean (standard deviations (SD)) and range [minimum - maximum]. Bold values correspond to significant statistical tests. Worst clinical and cognitive performances are characterized by a high repeat of CAG trinucleotides (CAG), high scoring on the Total Motor Score (TMS), and low scoring on the Total Functional Capacity (TFC), Verbal fluency (1 minute), Symbol digit modalities test (SDMT), Stroop color, Stroop word, Stoop interference.

Among the 137 participants, 131 were assessed for psychiatric symptoms with the PBA. **Table 3**. describes the PBA sub-scales scores across groups. Higher scores were observed for the Depression, Apathy, and Obsessive/compulsive sub-scales as the disease stage advanced.

**Table 3:** Problem Behaviors Assessment sub-scale scores.

Group	HC (N=34)	HD (N=97)			Group comparison & post-hoc
HD ISS		HD ISS 0-1 (N=22)	HD ISS 2 (N=21)	HD ISS 3 (N=54)	
***Depression***					***0.006***
* Mean (SD)*	*2.324 (3.641)*	*4.591 (6.254)*	*3.143 (4.799)*	*6.278 (5.941)*	*ISS 3 > HC*
* Range*	*0.000 - 16.000*	*0.000 - 19.000*	*0.000 - 19.000*	*0.000 - 24.000*	
***Irritability/aggressivity***					*0.823*
* Mean (SD)*	*0.441 (2.232)*	*0.636 (1.497)*	*0.810 (1.436)*	*0.778 (1.645)*	
* Range*	*0.000 - 13.000*	*0.000 - 6.000*	*0.000 - 4.000*	*0.000 - 8.000*	
***Apathy***					***0.004***
* Mean (SD)*	*0.206 (0.770)*	*0.182 (0.853)*	*0.000 (0.000)*	*1.537 (3.149)*	*ISS 3 > HC* *ISS 3 > 2*
* Range*	*0.000 - 4.000*	*0.000 - 4.000*	*0.000 - 0.000*	*0.000 - 16.000*	
***Obsessive/compulsive***					***≦ 0.001***
* Mean (SD)*	*0.059 (0.343)*	*0.000 (0.000)*	*0.286 (0.956)*	*2.741 (3.599)*	*ISS 3 > HC* *ISS 3 > ISS 0-1* *ISS 3 > ISS 2*
* Range*	*0.000 - 2.000*	*0.000 - 0.000*	*0.000 - 4.000*	*0.000 - 16.000*

Healthy controls (HC) and Huntington’s disease (HD) gene carriers were grouped according to the Integrating Staging System (ISS). The Problem Behaviors Assessment (PBA) sub-scales are calculated as the sum of the product of each items’ severity and frequency. An increase in PBA sub-scales characterizes worst psychiatric symptoms. The Depression sub-scale ranges from 0 to 48, Irritability/aggressivity and Obsessive/compulsive sub-scales from 0 to 32, and Apathy from 0 to 16. The scores’ mean (standard deviations (SD)) and range [minimum - maximum] are displayed. Bold values correspond to significant statistical tests.

### 3.2 Descriptive content of the emoHD corpus

The duration in seconds of the participants’ speech recordings in the emoHD corpus, excluding periods when the neuropsychologist was providing instructions or speaking, is shown in **Table 4**. The mean duration was similar across groups for the total recording duration, whereas the speech rate was lower for HD participants in the ISS 2 and 3 clinical stages compared to HC; and compared to HD ISS 0-1. Duration of the speech tasks can be found in the **Supplementary Table 2**.

**Table 4:** Speech recordings in seconds.

Group	HC (N=35)	HD (N=102)			Group comparison & post-hoc
HD ISS		0-1 (N=23)	2 (N=25)	3 (N=54)	
**Total (seconds)**					0.308
Mean (SD)	300.429 (145.562)	324.856 (102.775)	328.477 (218.957)	261.882 (187.862)	
Range	76.227 - 734.258	74.174 - 503.843	88.027 - 1109.034	28.829 - 981.835	
**Words/seconds**					**< 0.001**
Mean (SD)	2.704 (0.377)	2.714 (0.447)	2.287 (0.515)	2.009 (0.600)	*HC > ISS 2* *HC > ISS 3* *ISS 0-1 > ISS 2* *ISS 0-1 > ISS 3*
Range	1.954 - 3.527	2.065 - 3.954	1.352 - 3.388	0.278 - 3.111	

Speech task duration of healthy controls (HC) and Huntington’s Disease (HD) gene carriers grouped according to the Integrating Staging System (ISS). The duration is displayed in seconds with the mean [standard deviations (SD)] and range [minimum - maximum]. Bold values correspond to significant statistical tests.

**Figure 2:**
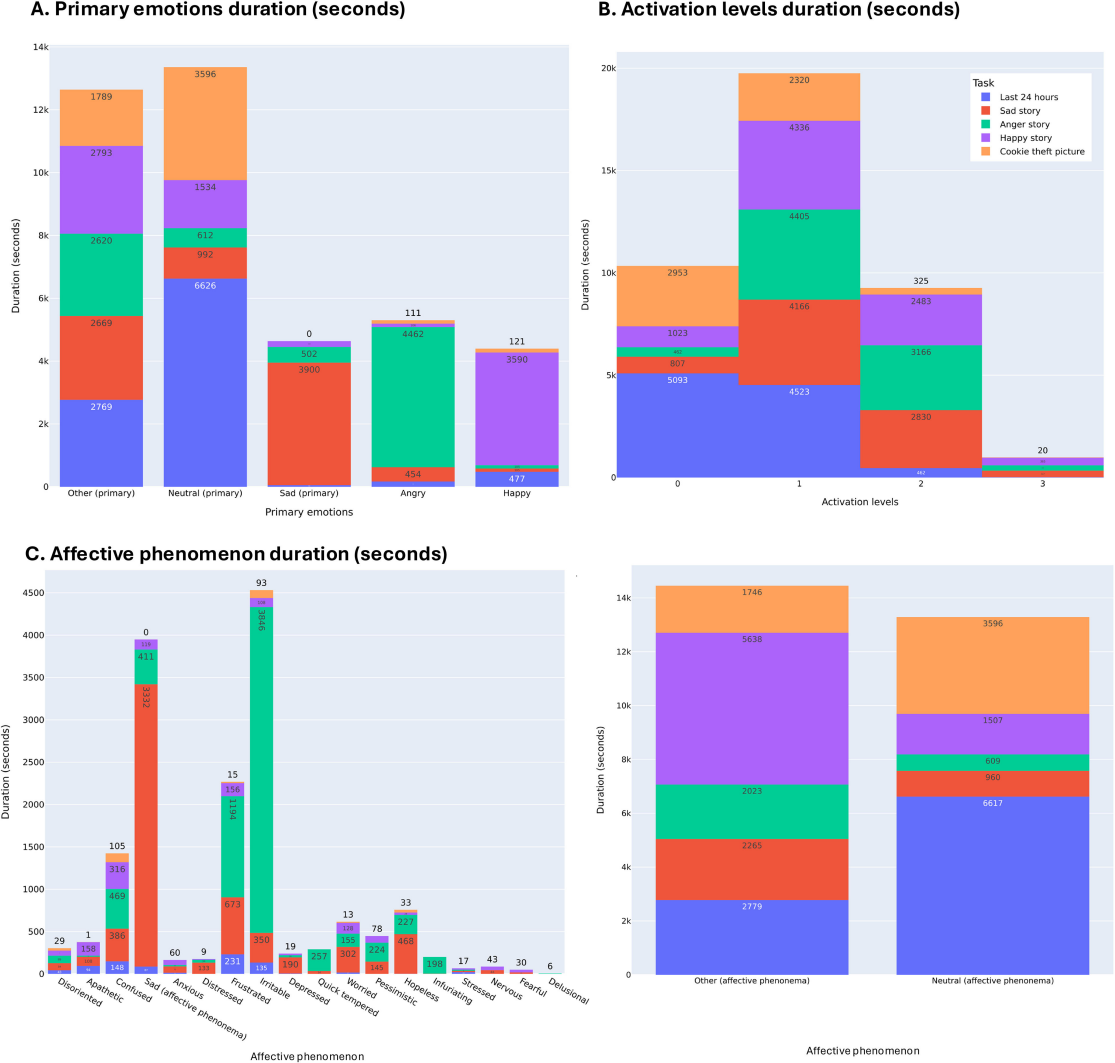
Distribution in seconds of primary emotions, affective phenomena and activation levels across the speech tasks. Distribution of primary emotions **(A)** and activation levels **(B)** across speech tasks. The distribution of affective phenomena is displayed separately for scaling reasons: neutral and other affective phenomena [Panel **(C)** right] and all other affective phenomena [Panel **(C)** left].

Annotating emotions took approximately 30 minutes per participant’s recordings. Once annotated, the emoHD corpus contained 6965 emotional segments defined by the speech therapists, which were labeled for primary emotions, affective phenomena and activation levels. The average emotional segment was 5.79 seconds, with a standard deviation of 6.17 and a range varying from 0.13 to 68.01 seconds. **Figure 2** displays the distribution (in seconds) of each possible label per speech tasks included in the emoHD corpus. The annotation of affective phenomena *violent, obsessive and lost* never occurred in the corpus.

### 3.3 Association between emotional descriptors

We obtain a mapping between annotated primary emotions, affective phenomena, and activation levels by evaluating their associations. This created a comprehensive understanding of their co-occurrence and validated the annotation scheme by demonstrating consistency between the labels of the emotional descriptors.

**Figure 3:**
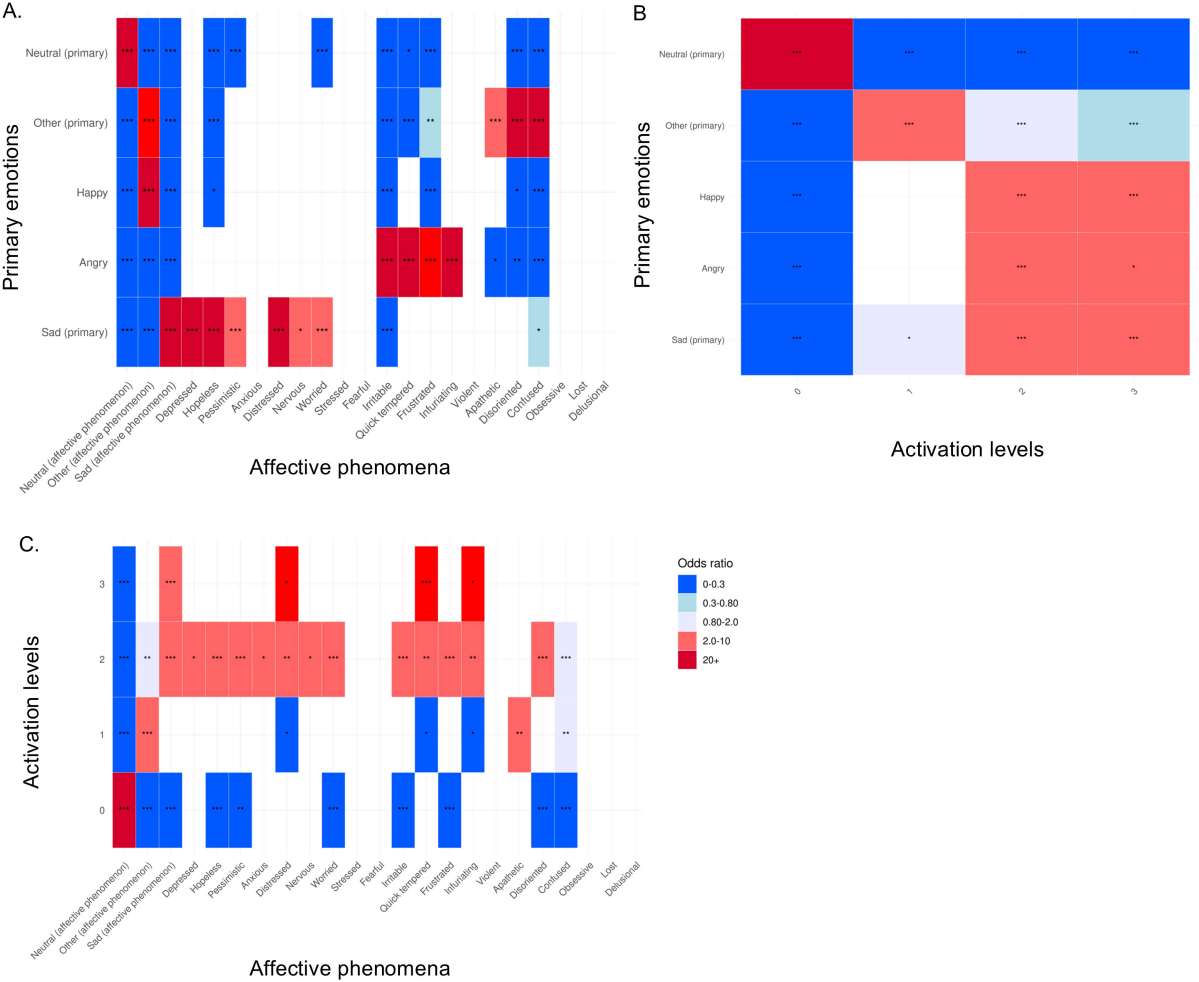
Heatmap of adjusted p-values of pairwise Fisher’s exact tests with Bonferroni multiple comparison correction. **(A)** Mapping between primary emotions and affective phenomena labels; **(B)** Mapping between primary emotions and activation levels labels; **(C)** Mapping between activation levels and affective phenomena labels. Significant adjusted p-values are displayed by *,**,*** corresponding to p-values equal to 0.05, 0.005, ≤0.001.

The Fisher exact tests examined the overall association between the categorical variables: primary emotions with affective phenomena, primary emotions and activation levels, and lastly affective phenomena with activation levels. All obtained a p-value (p) ≦ 0.001 after 10000 simulations with replicates. Each emotional descriptor had more than two labels, so the OR could not be calculated. Thus, the pairwise Fisher exact tests with multiple comparison correction mapped whether two labels were significantly associated with each other, with their OR indicating if there is no association (OR = 1), a positive association (OR > 1) or a negative association (OR < 1) (**Figure 3**).

As expected, there was a strong positive association of the primary emotion *neutral* with the affective phenomena *neutral* and activation level 0 (p ≦ 0.001, OR ≧ 20). Similarly, the affective phenomena consistently co-occurred with their related primary emotions that constituted a lower characterization of emotional experience, relating as a foundational component for more complex affective behaviors that are affective phenomena. The strength of positive associations of primary emotion *sad* was strong with affective phenomena *sad*, *depressed*, *hopeless*, *distressed* (p ≦ 0.001, OR ≧ 20), mild for *pessimistic* and *worried* (p ≦ 0.001, OR between 2 and 10), and weak for *nervous* (p ≦ 0.05, OR between 2 and 10). The mapping with *angry* displayed a strong positive association with *irritable, quick tempered*, and *infuriating* (p ≦ 0.001, OR ≧ 20), and mild for *frustrated* (p ≦ 0.001, OR between 10 and 20). The primary emotion *other* was strongly and positively associated with *disoriented*, and *confused* (p ≦ 0.001, OR ≧ 20), mildly with *other (affective phenomena)* and weakly with *apathetic* (p ≦ 0.001, OR between 2-10). Lastly, As the PBA assessed psychiatric symptoms and does not have any positive valence, *happy* was only associated positively with the affective phenomena *other* (p ≦ 0.001, OR ≧ 20).

Activation levels equal to 1 were mildly and positively associated with the primary emotion *other (*p ≦ 0.001, OR between 2-10). Regarding activation levels 2 and 3, mild positive associations were found with *sad, angry, and happy* (p ≦ 0.001, OR between 2-10).

Lastly, the mapping displayed multiple negative associations between the labels, which illustrated that they do not occur simultaneously in the emoHD corpus (OR between 0 and 0.80) (for example the primary emotion *happy* does not occur with the affective phenomena *irritable* (p ≦ 0.001, OR ≦ 1).

### 3.4 Emotional expression in Huntington’s disease gene carriers and healthy controls

#### 3.4.1 Primary emotions

**Figure 4:**
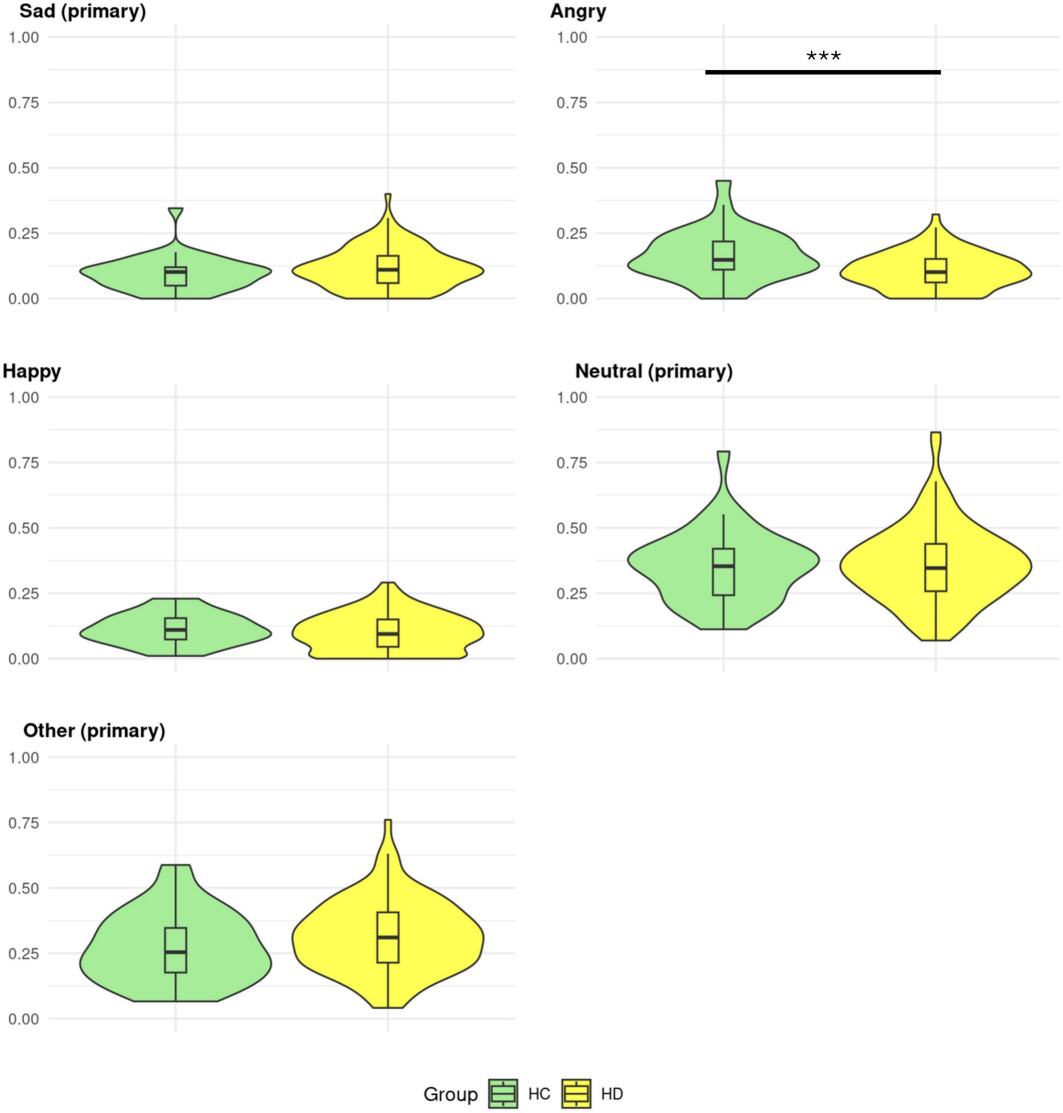
Proportion of primary emotions in Huntington’s disease gene carriers and controls. Healthy controls (HC) and Huntington’s disease (HD) gene carriers’ distributions of primary emotions proportions. The proportions range from 0-1. Significant group differences are displayed by *,**,*** corresponding to permutation p-values equal to 0.05, 0.005, ≤0.001.

HD gene carriers and HC differed regarding their proportion of expressed *angry* (t-statistic (t) = 3.46 (51.12), p = 0.001, permutation p-value (perm-p) ≦ 0.001) with HC exhibiting higher (( $\bar{x}$ ) = 0.165) proportions *angry* than HD ( $\bar{x}$ = 0.103) (**Figure 4**). Tukey’s test revealed that this was carried by gene carriers in the HD ISS stage 3 exhibiting a lower proportion than the HC individuals (estimate = -0.07, p ≤ 0.001). The remaining proportions of primary emotions were similar across HD gene carriers and HC (all p ≧ 0.084, perm-p ≧ 0.108): proportion of *sad* (t = -1.75 (83.07)), *happy* (t = -1.50 (84.03)), *neutral* (t = -0.47 (75.26)) and *other (*t = -1.54 (70.67)). See **Supplementary Table 3** for details.

#### 3.4.2 Affective phenomena

**Figure 5:**
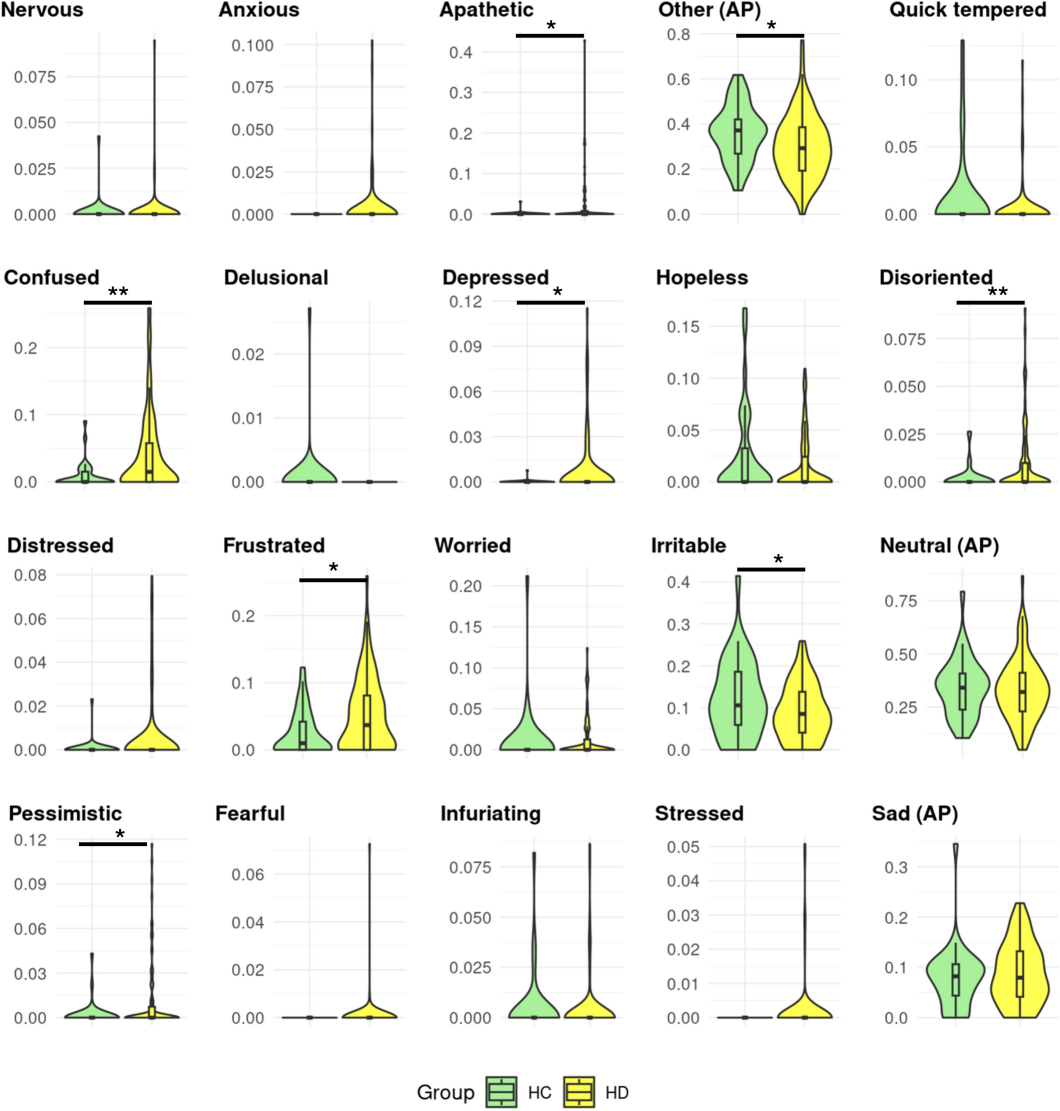
Proportions of affective phenomena in Huntington’s disease gene carriers and controls. Healthy controls (HC) and Huntington’s disease (HD) gene carriers’ distributions of affective phenomena (AP) proportions. The proportions range from 0-1. Significant group differences are displayed by *,**,*** corresponding to permutation p-values equal to 0.05, 0.005, ≤0.001.

The emoHD corpus participants expressed some affective phenomena differently (**Figure 5**). Pairwise group difference assessed by the Tukey’s test based on the HD ISS revealed which groups carried the difference.

The proportion of *apathetic (*HD $\bar{x}$ = 0.024, HC = $\bar{x}$ = 0.001, t = -3.16 (77.22), p = 0.002, perm-p = 0.025) and *depressed (*HD $\bar{x}$ = 0.007, HC $\bar{x}$ = 0.000, t = -2.82 (76.25), p = 0.006, perm-p = 0.049) were higher in HD gene carriers than HC, with no specific pairwise group differences (all p ≧ 0.63).

The proportions of *confused (*HD $\bar{x}$ = 0.043, HC $\bar{x}$ = 0.009, t = -4.17 (99.62), p ≦ 0.001, perm-p = 0.003), *disoriented (*HD $\bar{x}$ = 0.011, HC $\bar{x}$ = 0.002, t = - 3.50 (102.32), p ≦ 0.001, perm-p = 0.011) were higher in HD gene carriers than HC: HD ISS 3 having higher proportions than HC (*confused* estimate *=* 0.040, p = 0.004; *disoriented* estimate = 0.001, p = 0.041). *Pessimistic* was also higher in HD (HD $\bar{x}$ = 0.012, HC $\bar{x}$ = 0.002, t = - 3.20 (99.32), p = 0.002, perm-p = 0.020), HD ISS 2 having higher proportions than HC (estimate = 0.02, p = 0.033). Proportions of expressed *frustrated* were higher in HD than HC (HD $\bar{x}$ = 0.048, HC $\bar{x}$ = 0.025, t = - 2.70 (100.03), p = 0.008, perm-p = 0.022) due to HD ISS 0–1 carrying these higher proportions compared of HC (estimate= 0.04, p = 0.018).

Proportions of *other* (HD = 0.295, HC = 0.360, t = 2.48 (78.09), p = 0.016, perm-p = 0.021) and *irritable* (HD = 0.085, HC = 0.121, t = 2.08 (50.58), p = 0.043, perm-p = 0.020) were lower in the HD group than HC. HC had higher proportions than the HD ISS 3 for both these affective phenomena (*other* estimate = - 0.08, p = 0.05; *irritable* estimate = - 0.04, p = 0.394).

No other affective phenomena displayed a group effect when comparing HD gene carriers and HC (all p & perm-p ≧ 0.05). See **Supplementary Table 4** for details.

#### 3.4.3 Activation

**Figure 6:**
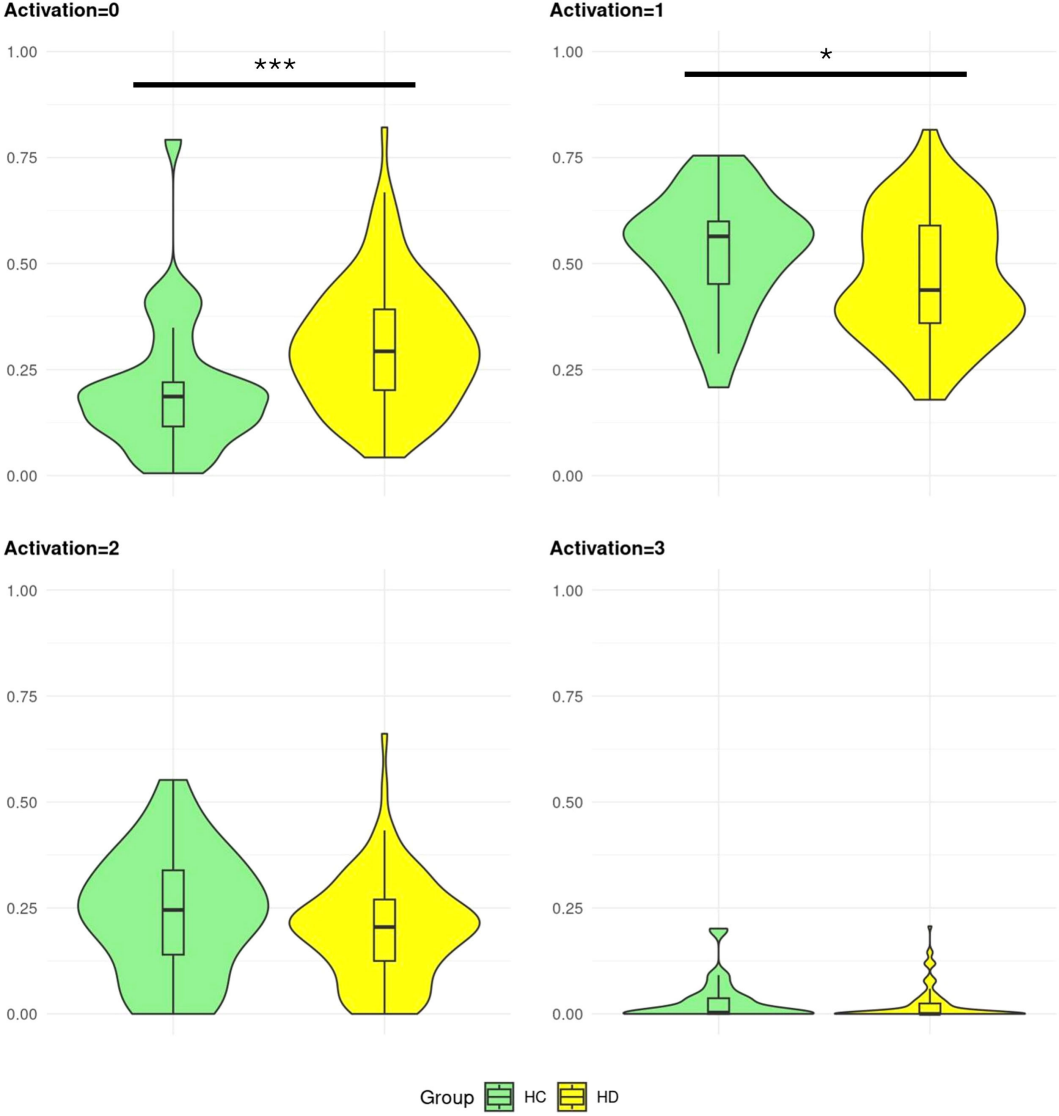
Proportion of activation levels in Huntington’s disease gene carriers and controls. Healthy control (HC) and Huntington’s disease (HD) gene carriers’ distributions of activation levels proportions. The proportions range from 0-1. Significant group differences are displayed by *,**,*** corresponding to permutation p-values equal to 0.05, 0.005, ≤0.001.

HD gene carriers showed higher proportions of activation levels equal to 0 than HC (HD = 0.313, HC = 0.203, t = -3.59 (64.22), p & perm-p ≦ 0.001), carried by HD ISS 3 and 2 having higher proportions than HC (respectively estimate = 0.12, p = 0.002; estimate = 0.12, p = 0.138) (**Figure 6**). HD gene carriers exhibited lower proportions of activation levels equal to 1 than HC (HD = 0.472, HC = 0.530, t = 2.09 (71.56), p = 0.040, perm-p = 0.045), with no specific pairwise differences between HD ISS groups and controls (p ≧ 0.148). There were no other differences in activation levels (p ≧ 0.144, perm-p ≧ 0.126): activation equal to 2 with t = 1.47 (59.52) and equal to 3 with t = 1.19 (47.06). See **Supplementary Table 5** for details.

### 3.5 Associations between emotions and psychiatric behaviors

**Figure 7:**
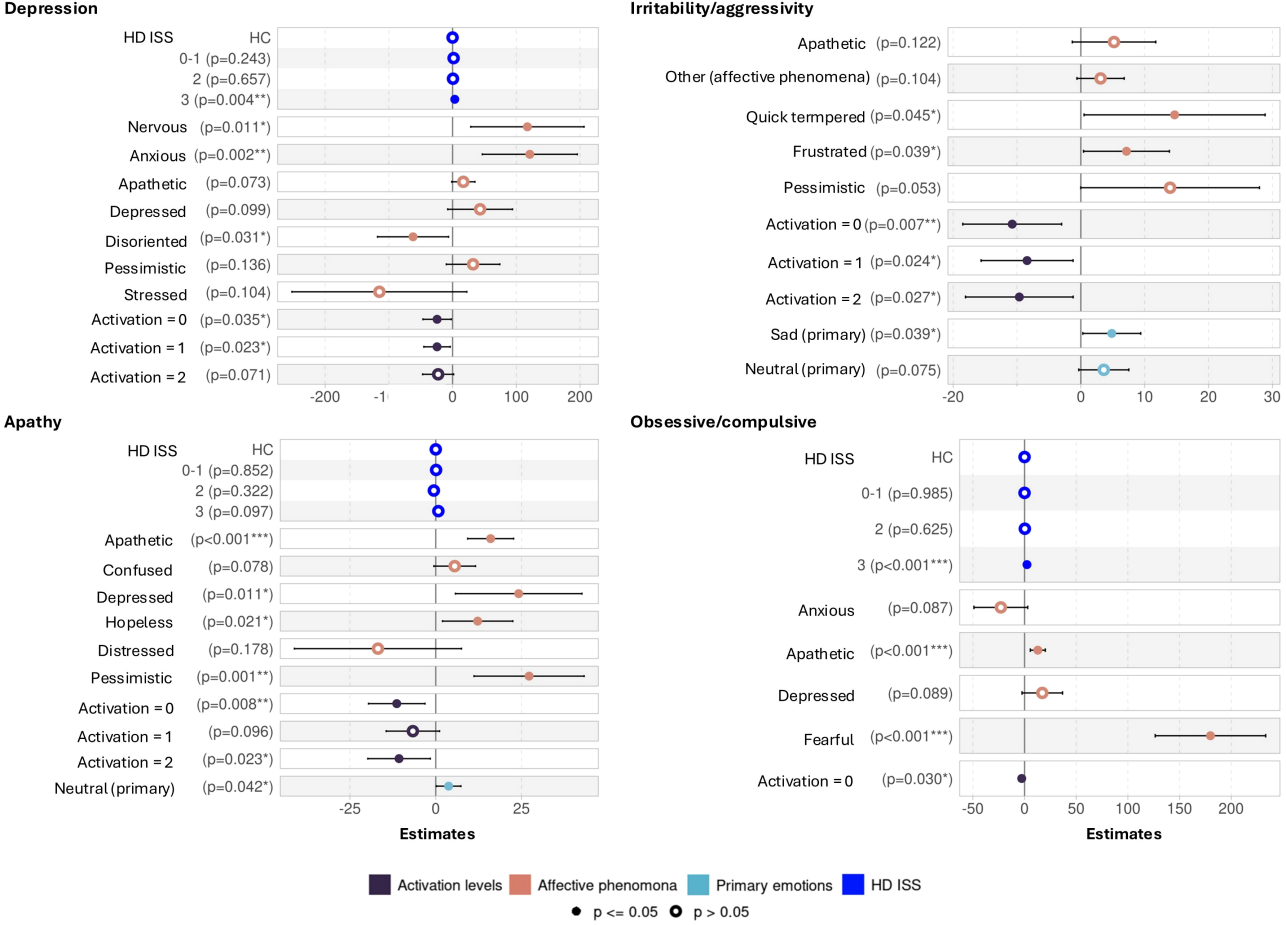
Multilinear regressions after a stepwise bidirectional approach for PBA sub-scales. Healthy controls (HC) and Huntington’s Disease (HD) gene carriers grouped according to the Integrating Staging System (ISS). The figure displays the results of the multilinear regressions with stepwise bidirectional approach, evaluating the associations between the different emotional descriptors (primary emotions, affective phenomena, and activation levels) and the Depression, Irritability/aggressivity, Apathy and Obsessive/compulsive Problem Behaviors Assessment (PBA) sub-scales. An increase in PBA sub-scales characterizes worst psychiatric symptoms. The Depression sub-scale ranges from 0 to 48, Irritability/aggressivity and Obsessive/compulsive sub-scales from 0 to 32, and Apathy from 0 to 16. The emotional descriptors are expressed in proportions normalizing total speech duration. The bidirectional stepwise approach selected the emotional descriptors that best fit the PBA sub-scales, and displaying the significantly associated ones by a filled dot and by *,**,*** corresponding to p-values equal to 0.05, 0.005, ≤0.001.

Multilinear regressions evaluated the associations between the proportions of emotional descriptor labels with each PBA sub-scale score (**Figure 7**). Using a stepwise bidirectional approach, we determined which important emotional descriptor labels were associated with psychiatric scores, adjusting for sex, age and the clinical group status (HD ISS 0-1, 2, 3 and HC).

The expression of affective phenomena *anxious* (p = 0.001, confident interval (CI) = 46.63 – 195.73, estimate = 121.18) and *nervous* (p = 0.010, CI = 28.53 – 206.28, estimate = 117.40) were positively associated to the Depression sub-scale (R2 = 0.317). In other words, greater expression of these affective phenomena is associated with a higher Depression sub-scale score. On the other hand, higher expression of the affective phenomena *disoriented* appeared to be preventive for Depression (p = 0.031, CI = -117.72 – -6.32, estimate = -62.02). Depression appeared slightly associated with HD ISS 3 (p = 0.004, CI = 1.14 – 5.85, estimate = 3.49).

Irritability/aggressivity sub-scale (R2 = 0.190) was positively associated with the affective phenomenon *quick tempered and frustrated* (respectfully p = 0.045, CI = 0.51 – 28.87, estimate = 14.69; and p = 0.037, CI = 0.42-13.87, estimate = 7.14), and the primary emotion *sad* (p = 0.039, CI = 0.30 – 9.39, estimate = 4.84) without any association with the HD ISS status.

The Apathy sub-scale (R2 = 0.414) was strongly and positively associated with the affective phenomena *pessimistic* (p = 0.001, CI = 11.15 – 43.25, estimate = 27.20), followed by *depressed* (p = 0.010, CI = 5.73 – 42.62, estimate = 24.17)*, apathetic* (p ≦ 0.001, CI = 9.29 – 22.69, estimate = 15.99) and *hopeless (*p = 0.001, CI = 1.97 – 22.46, estimate = 12.22), and weakly with the primary emotion *neutral* (p = 0.040, CI = 0.17 – 7.32, estimate = 3.75). No effect of the HD ISS status was identified.

The Obsessive/compulsive sub-scale (R2 = 0.512) was associated positively with the affective phenomena *fearful (*p ≦ 0.001, CI = 126.44 – 233.37, estimate = 179.90) followed by *apathetic* (p = 0.001, CI = 5.58 – 20.11, estimate = 12.84), and with the HD ISS stage 3 (p ≦ 0.001, CI = 1.45 – 3.26, estimates = 2.35).

Across the four multilinear models, we observed weak to moderate negative associations between the scales and the activation levels 0, 1, and/or 2 (p ≦ 0.05) indicating that individuals with higher psychiatric symptoms tended to exhibit lower excitation levels in their voice. We also see that the highest level of activation (equal to 3) was not associated with any sub-scale. Detailed results are available in the **Supplementary Table 6**–**9**.

## 4 Discussion

We explored the connection between emotional expression and psychiatric symptoms in HD by developing the first annotated HD emotional speech corpus, emoHD, and assessed its congruence with the PBA. By comparing HD gene carriers and HC, we found that HD individuals exhibited slightly more Depression, Apathy and Obsessive/compulsive symptoms on the PBA sub-scales, but surprisingly similar to controls regarding Irritability/aggressivity. HD speech contained a greater proportion of segments with no emotional activation (level 0), while fewer segments with mild emotional activation (level 1), denoting fewer emotional expressions than controls. HD gene carriers and controls did not differ regarding their expression of primary emotions, except for a lower expression of *angry* in HD, primarily due to patients in later disease stages. In contrast, HD gene carriers expressed more the affective phenomena *apathetic*, *confused, depressed, disoriented, frustrated, and pessimistic* than controls, while expressing less *other* and *irritable*. This suggests that the affective phenomena framework is more appropriate to capture emotional speech in HD in comparison to primary emotions. Altogether, we demonstrated for the first time, a congruence between the emotional expressions in speech and psychiatric symptoms in HD, revealing which specific emotional expressions are indicative of an increase in the PBA sub-scale scores (**Table 5**).

**Table 5:** Summary of main findings.

A. Emotional expression in speech			
Differences	Primary emotions	Affective phenomena	Activation levels
HD > HC		*apathetic* *confused* *depressed* *disoriented* *frustrated* *pessimistic*	*level 0 (=neutral)*
HD < HC	*angry*	*irritable* *other*	*level 1 (=mild)*
B. Positive associations with the PBA sub-scales			
PBA sub-scale	Primary emotions	Affective phenomena	
Depression		*anxious* *nervous*	
Apathy	*neutral*	*pessimistic* *depressed* *apathetic* *hopeless*	
Irritability/aggressivity	*sad*	*quick tempered* *frustrated*	
Obsessive/compulsive		*fearful* *apathetic*	

The table displays in A. which emotional descriptors (primary emotions, affective phenomena, and activation levels) differ in Huntington’s Disease (HD) gene carriers compared to healthy controls (HC): > for HD expressing them more than HC, and < for less. In B. the table displays the found positive associations between the emotional descriptors and the Depression, Irritability/aggressivity, Apathy and Obsessive/compulsive Problem Behaviors Assessment (PBA) sub-scales: if the emotional descriptor increases then the sub-scales does too.

One might be surprised that *angry* was less expressed in HD patients’ speech than in controls. However, previous research on the same cohort has shown that, when assessing the general emotional content of emotional speech tasks, patients narrating anger and happy stories exhibited lower emotional content compared to healthy individuals (67). This lower expression of *angry* aligns with HD’s consistently reported difficulty in recognizing facial and vocal emotional expressions of anger (68–71), as well as the observed tendency to be less capable of facially imitating anger when prompted by a card (72). Our analysis of emotional segments of HD patient’s speech, showed that they not only struggle in recalling and narrating anger-charged stories but also express that emotion less. Furthermore, in our study, patients spoke more without emotional activation (level 0) and less at mild emotional activation (level 1) than controls, showing a general diminished capacity for oral emotional expression. This might be linked to the dysprosody in HD where patients may struggle to convey emotions through vocal tone, pitch and prosodic variation (5, 73, 74). Patients’ dysarthria, a motor disorder affecting speech clarity, articulation, vocal control and speech rate (75), may also contribute to decreasing the effectiveness of their communication. However, research on patients without buccofacial motor disorders suggests that oral and facial emotional production deficits are not solely due to motor impairments, but also involve complex cognitive, neuropsychiatric and emotional processing deficits (3, 7, 72, 75).

In contrast, patients spontaneously expressed many emotions not elicited by the emotional-speech tasks (e.g. sad, angry and happy), captured under the emotional descriptor affective phenomena. These were more nuanced than Ekman’s universal emotions, which have been criticized for being too rigid and simplistic to capture the complexity of real-life emotional experiences (76). We addressed this challenge by selecting the affective phenomena labels as listed by external participants exposed to PBA statements, to minimize potential clinical and research bias. Unlike other emotional speech corpora featuring pathological speakers (21, 22, 37, 39), we focus on emotional states reflecting potential disease-specific manifestations, rather than on basic, coarse-grained emotions. In this context, affective phenomena provided a clearer way to identify predominant emotional behaviors in HD such as *confusion, disorientated*, and *pessimistic*. These became more pronounced as the disease progressed, particularly in HD ISS 2 or 3, which is congruent with the documented increase in obsessive-compulsive and disorientation PBA item scores across HD stages (77). *Apathetic* and *depressed* expressions were similar across disease stages, suggesting that these may be experienced throughout the disease course as affective dispositions or moods [i.e., personality disposition or an episodic state as defined by Scherer (43)]. This was the case regardless of a formal diagnosis of depression or apathy which occurred in later disease stages in the emoHD cohort. We further found that gene carriers without any motor or cognitive symptoms (ISS 0-1) expressed more *frustrated*, presumably due to the anticipated disease progression and the negative impact of a recent diagnosis. This negative impact has been shown to affect multiple psychiatric symptoms in HD, even before onset of the disease (78). Surprisingly, HD patients exhibited less *irritability* than controls, consistent with the lack of group difference on the Irritability/aggressivity sub-scale. One possibility is that patients were genuinely not irritable at the time of the interview, as those experiencing irritability may have opted out of the speech recordings. Another possibility is that patients’ behavior in the hospital setting may differ from their behavior at home.

Limited research has explored the interplay between emotions and psychiatric behaviors in HD. Existing studies consistently reported associations between apathy - the most prevalent psychiatric symptom in HD (79) - and impaired facial emotion recognition, typically assessed using tasks that evaluate Ekman’s emotions (80–82). Our study included primary emotions but extended the scope of investigation by introducing affective phenomena and examining their relationships with a wider range of psychiatric symptoms. Alternative approaches have linked HD psychiatric symptoms to self-reported emotional well-being assessed with quality of life scales, showing that lower emotional well-being correlated with more severe symptoms (83, 84). However, quality of life scales may limit the set of emotional questions, restricting patients’ from naturally expressing their emotions and feelings, and therefore failing to accurately capture their emotional state (85). Spontaneous speech is a more ecologically valid method because it can directly link the expressed emotions cross-sectionally with the patient’s current psychiatric well-being.

Although corpora from healthy individuals have been used to develop speech emotion recognition models for conditions like Alzheimer’s and Parkinson’s disease (39–41), neurological disorders include unique language, speech, cognitive and emotional deficits, thus, making the use of healthy individuals’ data insufficient to fully represent the profiles of the targeted neurological conditions (86). The emoHD corpus followed the principles outlined by Douglas-Cowie for creating a high-quality and generalizable emotional speech corpus (87): scope of speakers, naturalness of speech, context and emotional descriptors. Indeed, EmoHD targeted a large sample of genetically confirmed HD gene carriers with varied disease clinical stages, alongside control participants for comparison purposes. We prioritized elicited emotional speech to maximize the ecological validity of the corpus, having a trade-off between the limitations of acted emotional speech — which fails to convey real-life emotions (88) — and natural emotional databases — which often face legal and ethical issues (89, 90). The emotional speech tasks successfully elicited primary emotions, as evidenced by their predominance in their respective tasks, and allowed for the expression of non-elicited emotions, captured through affective phenomena (**Figure 2**). Regarding contextual information, our design included psychiatric assessments conducted by certified neurologists — an aspect often lacking in emotional speech corpora with neurological patients as speakers. Indeed, such corpora prioritize cognitive or motor assessments over psychiatric insights (36, 38, 90, 91), or rely on self-reported psychiatric scales that may not accurately reflect the patient’s psychiatric state (35, 37, 92). Lastly, by annotating emotions and quantifying their duration within patients’ speech, we captured emotion-carrying segments of speech. In contrast, prior work on HD emotional expression (5, 67) typically assumed that the emotion elicited remained present throughout the entire recording, using it as the emotional label. While eliciting emotions (93) and successful narration must arouse emotion in speakers (55, 56), these approaches implied that emotional expression remains constant throughout the narrative speech— which was rarely the case in the annotated emoHD corpus.

Speech appears as a non-invasive marker of HD patients’ emotional well-being and psychiatric status, complementing its established use in the assessment of motor, functional and cognitive performance (52, 94–99). More generally, speech has been recognized as an insightful marker for psychological and psychiatric monitoring. Alteration in speech signals (e.g., prosodic, rhythm changes, etc.) (28, 29, 100) and lexical and semantic patterns (101) are markers of various conditions. This includes mood disorders such as depression and anxiety, psychotic disorders, or emotional processing deficits like alexithymia. Using speech as a non-invasive objective marker has encouraged the development of machine learning pipelines that analyze speech to detect and monitor mental health conditions (20, 28). By analyzing an individual’s speech frequently, these models could help clinicians adapt personalized treatment plans, detect early warning signs, and deliver targeted interventions based on speech-derived markers.

The annotation and validation of the emoHD dataset in our study makes it now possible to use it to train speech emotion recognition (SER) models. These models are designed to identify expressed emotions based on the speakers’ acoustic signal (102). In the case of HD, a SER model could enable the automatic annotation of patients’ speech, providing a scalable tool to detect emotional disturbances. This output can then be further used to predict their psychiatric behaviors. As our speech recordings ranged from 4.3 to 5 minutes, this approach would be notably less time-consuming than a full PBA evaluation, which typically lasts around 20 mins (103). Hence integrating a SER model into HD clinical care would support the remote monitoring of emotional and psychiatric symptoms. This would also assist in the early detection of behavioral changes and track the progression of these symptoms and thus enhance personalized medicine. Given the challenge many HD patients face in accessing healthcare systems — such as medical experts, medical deserts and long appointment delays (104–106) — speech, which is easy to collect, would allow to perform additional clinical assessment of patients’ in their homes, in-between physical medical appointments.

Some limitations and challenges should be considered in future work. First, although we included 102 HD gene carriers, the sample size remains restrained for normative data in a neurodegenerative disease. Nevertheless, our sample size remains acceptable for a rare disease as HD, and our cohort is larger than most existing emotional speech corpus with pathological speakers (35, 41), providing a valuable contribution in a field where datasets are often limited. As emoHD is an elicited emotional dataset, the recording conditions ensured consistency across participants, minimizing background noise and language-related confounds. This allowed us to reliably isolate disease-specific emotional markers of psychiatric symptoms. Second, in the present study, emotional annotation remained a time-consuming process (30 minutes per recording). However, now that the emoHD corpus has been annotated, it can be used to train a SER model, enabling future recordings to be automatically annotated without the need for manual annotations. Third, while we were able to explore the associations between emotional expressions and psychiatric behaviors, our cohort suffers the same bias as all HD cohorts, namely not including individuals at high-risk of engaging in harmful behaviors towards themselves or others, as urgent care is of higher priority than volunteering to partake in studies. Apart from the Irritability/aggressivity sub-scales that did not differ between HD ISS and controls, the scores on the PBA sub-scales remained similar to other cohorts like REGISTRY HD (107). To account for these limitations, we enhanced our statistical power by applying permutation based statistical tests (63, 64), and incorporating healthy controls with some psychiatric symptoms. Additionally, *post-hoc* analysis of possible cofounders of speech have been evaluated (**Supplementary Table 10**), suggesting that language cognitive abilities, educational level and antipsychotic drugs influence HD participants total speech time, which should be taken into consideration in future studies. Lastly, regarding the valence of our different emotional descriptors’ labels, we are aware that they are majorly negative. Indeed, the affective phenomena were chosen based on external participants’ interpretations of the PBA, which lacks any positive statements. We anticipated that the primary emotion *happy* would either not show any associations, negatively correlate with most affective phenomena or be positively associated with the label *other*, which was the case when we analyzed the emotional descriptors associations. As our aim is not to evaluate patients’ quality of life or positivity, we acknowledge this limitation, which does not significantly impact our approach in the exploration of emotions and their associations to psychiatric behaviors.

## 5 Conclusion

By developing the emoHD corpus, we were able to associate emotional expression and psychiatric behaviors in Huntington’s Disease underscoring the relevance of our findings in this population. This work encourages future work to specifically develop speech-based digital tools that can assess Huntington’s Disease patients emotional and psychiatric well-being. HD serves as an excellent model for studying neurodegenerative disorders (108) because of the multifaceted nature of motor, cognitive, and psychiatric symptoms and their interplay. We further propose that the emoHD framework’s flexibility and adaptability can be developed and applied in other neurodegenerative diseases with affective disorders, by applying the proposed method to the collected speech samples. This would identify other disease-specific emotional and psychiatric associations.

## Data Availability

The conditions of our ethics approval, which includes the ethical consent of participants and the collected data, do not allow for public archiving of anonymized study data. On reasonable request, readers seeking access to the data should contact the corresponding author. Access will be granted to named individuals in accordance with ethical procedures for the reuse of sensitive data, including a research partnership and completion of a data transfer partnership by the APHP. Legal copyright restrictions prevent public archiving of the UHDRS, which can be obtained from UHDRS® | - Huntington Study Group, which can be obtained from the agency pour la protection des programmes IDDN FR 001 230010 001 S A 2020 000 31 230. Statistical analysis script can be found on https://gitlab.cognitive-ml.fr/lchenain/emohd_corpus.git.
