# Supplementary material for: Emotional speech markers of psychiatric disturbance in Huntington’s disease
Source: Front Psychiatry. 2025 Aug 12;16:1633492. doi: 10.3389/fpsyt.2025.1633492 (PMC12378116; doi:10.3389/fpsyt.2025.1633492)
Supplement: Supplementary file 1 [file Table1.docx]

Supplementary Material

**Table 1. Naive participants' demographic information**

*Demographic information of external participants. Huntington’s Disease (HD); Problem Behaviors Assessment (PBA)*

|  | **Participants (n=96)** |
| --- | --- |
| Age (years) | 35.61 years (± 15.17) [19.0 ; 79.0] |
| Genders | 76.0% Female 21.9% Male  1.0 % Other  1.0 % Not specified |
| Education | 1.0 % No schooling complete  1.0 % Professional aptitude certificate  4.2 % High school graduate  6.2 % Associate degree (2-years)  15.6 % Bachelor’s degree (3 or 4-years) 51.0 % Master’s degree (5-years)  20.8 % Professional degree (8-years) |
| Clinical activity | 89.6 % Not at all 4.2 % A little  4.2 % Moderately  1.0% A lot  1.0% Completely |
| Familiar with HD | 74.0 % Not at all  17.7 % A little  6.2 % Moderately  2.1 % A lot  0.0 % Completely |
| Familiar with the PBA | 92.7 % Not at all  6.2 % A little  0 % Moderately  0 % A lot  1 % Completely |

**Table 2. Speech recordings in seconds across speech tasks**

*Speech task duration of healthy control participants (HC) and Huntington’s Disease (HD) gene carriers grouped according to the Integrating staging system (ISS). All speech task duration is displayed in seconds with the mean (standard deviations (SD)) and range [minimum - maximum].*

| ***Group*** | ***HC (N=35)*** | ***HD (N=102)*** | | | ***Group comparison & post-hoc*** |
| --- | --- | --- | --- | --- | --- |
| **HD ISS** |  | **0-1 (N=23)** | **2 (N=25)** | **3 (N=54)** |  |
| Last 24 hours task |  |  |  |  | **0.038** |
| Mean (SD) | 68.622 (33.402) | 90.563 (50.826) | 100.668 (132.719) | 57.825 (40.093) |  |
| Range | 25.856 - 173.131 | 0.000 - 242.130 | 32.589 - 717.034 | 0.000 - 243.430 |  |
| Sad story task |  |  |  |  | 0.649 |
| Mean (SD) | 59.169 (43.154) | 67.416 (33.477) | 60.401 (38.077) | 53.926 (48.253) |  |
| Range | 0.000 - 205.274 | 14.500 - 160.387 | 12.623 - 159.177 | 0.000 - 223.670 |  |
| Anger story task |  |  |  |  | 0.684 |
| Mean (SD) | 65.012 (41.653) | 66.077 (32.053) | 60.554 (38.567) | 55.321 (51.057) |  |
| Range | 0.000 - 220.524 | 0.000 - 137.181 | 0.000 - 147.749 | 0.000 - 281.209 |  |
| CTP task |  |  |  |  | 0.807 |
| Mean (SD) | 39.814 (17.822) | 37.089 (17.619) | 42.774 (19.909) | 41.344 (25.439) |  |
| Range | 0.000 - 73.882 | 0.000 - 66.566 | 14.003 - 114.103 | 0.000 - 163.606 |  |
| Happy task |  |  |  |  | 0.896 |
| Mean (SD) | 63.686 (45.558) | 57.974 (35.058) | 54.754 (42.368) | 59.345 (49.674) |  |

**Table 3. Statistical analysis of primary emotions**

*Group differences in the proportions of primary emotion between healthy participants (HC) and Huntington’s Disease (HD) gene carriers with permutated t test, displaying p-values (p), permutation p-values (perm p), and if significant (perm p ≦0.05), permutation ANOVA (perm ANOVA) with Tukey post hoc (estimates (M), standard error (SE), t-value (t) and p-value (p)) according to the HD Integrated Staging System (ISS). Non-applicable (na)*

| **Proportion**  **of** | **t test** | **mean of HC** | **mean of HD** | **p** | **perm p** | **perm ANOVA** | **perm p** | **tukey** |
| --- | --- | --- | --- | --- | --- | --- | --- | --- |
| Sad | -1.75 (83.07) | 0.095 | 0.121 | 0.084 | 0.108 | na | na | na |
| Angry | 3.46 (51.12) | 0.165 | 0.103 | 0.001 | ≦0.001 | F(3,133)=5.75 | ≦0.001 | HD ISS 3 - HC : M=−0.06839,SE=0.01660,t=−4.119,p<0.001) |
| Happy | 1.50 (84.03) | 0.114 | 0.094 | 0.137 | 0.172 | na | na | na |
| Neutral | -0.47 (75.26) | 0.352 | 0.366 | 0.638 | 0.658 | na | na | na |
| Other | -1.54 (70.67) | 0.274 | 0.316 | 0.129 | 0.143 | na | na | na |

**Table 4. Statistical analysis of affective phenomena**

*Group differences in the proportions of affective phenomena between healthy participants (HC) and Huntington’s Disease (HD) gene carriers with permutated t test, displaying p-values (p), permutation p-values (perm p), and if significant (perm p ≦0.05), permutation ANOVA (perm ANOVA) with Tukey post hoc (estimates (M), standard error (SE), t-value (t) and p-value (p)) according to the HD Integrated Staging System (ISS). Non applicable (na)*

| **Proportion of** | **t test** | **mean of HC** | **mean of HD** | **p** | **perm p** | **perm ANOVA** | **perm p** | **tukey** |
| --- | --- | --- | --- | --- | --- | --- | --- | --- |
| Nervous | -0.21 (96.78) | 0.001 | 0.002 | 0.831 | 0.953 | na | na | na |
| Anxious | -2.06 (75) | 0.000 | 0.003 | 0.043 | 0.155 | na | na | na |
| Apathetic | -3.16 (77.22) | 0.001 | 0.024 | 0.002 | 0.025 | F(3,133)=2.26 | 0.101 | No significant pairwise group difference |
| Other | 2.48 (78.09) | 0.360 | 0.295 | 0.016 | 0.021 | F(3,133)=2.48 | 0.046 | HD ISS 3 - HC : M =−0.07637,SE=0.02942,t=−2.596,p=0.050 |
| Quick tempered | 1.316 (45.45) | 0.012 | 0.005 | 0.195 | 0.114 | na | na | na |
| Confused | -4.17 (99.62) | 0.009 | 0.043 | ≦0.001 | 0.003 | F(3,133)=4.59 | ≦0.001 | HD ISS 3 - HD : M=0.039784,SE=0.011477,t=3.466,p=0.0039 |
| Delusional | 1.00 (34.00) | 0.001 | 0.000 | 0.324 | 0.324 | na | na | na |
| Depressed | -2.82 (76.25) | 0.000 | 0.007 | 0.006 | 0.049 | F(3,133)=1.33 | 0.217 | No significant pairwise group difference |
| Hopeless | 1.17 (47.83) | 0.026 | 0.017 | 0.249 | 0.174 | na | na | na |
| Disoriented | -3.50 (102.32) | 0.002 | 0.011 | ≦0.001 | 0.011 | F(3,133)=2.80 | 0.021 | HD ISS 3 - HC : M=0.008925, SE= 0.003333,t= 2.678 ,p=0.0407 |
| Distressed | -1.91 (97.80) | 0.001 | 0.004 | 0.060 | 0.188 | na | na | na |
| Frustrated | -2.70 (100.03) | 0.025 | 0.048 | 0.008 | 0.022 | F(3,133)=3.15 | 0.050 | HD ISS 0-1 - HC : M=0.03934,SE=0.01326,t=2.967,p=0.018 |
| Worried | -0.31 (50.34) | 0.011 | 0.013 | 0.762 | 0.738 | na | na | na |
| Irritable | 2.08 (50.58) | 0.121 | 0.085 | 0.043 | 0.020 | F(3,133)=2.61 | 0.046 | HD ISS 3 - HC : M=-0.042260, SE= 0.015720,t=-2.688,p=0.0394 |
| Neutral | 0.17 (73.96) | 0.343 | 0.338 | 0.864 | 0.873 | na | na | na |
| Pessimistic | -3.20 (99.32) | 0.002 | 0.013 | 0.002 | 0.020 | F(3,133)=2.70 | 0.008 | HD ISS 2 - HC: M=0.015020, SE=0.005453,t=2.754,p=0.0332 |
| Fearful | -1.11 (75.00) | 0.000 | 0.001 | 0.272 | 0.787 | na | na | na |
| Infuriating | 0.98 (42.76) | 0.005 | 0.002 | 0.331 | 0.276 | na | na | na |
| Stressed | -1.69 (75.00) | 0.000 | 0.001 | 0.096 | 0.484 | na | na | na |
| Sad | -0.76 (66.03) | 0.079 | 0.089 | 0.449 | 0.449 | na | na | na |

**Table 5. Statistical analysis of activation levels**

*Group differences in the proportions of activation levels between healthy participants (HC) and Huntington’s Disease (HD) gene carriers with permutated t test, displaying p-values (p), permutation p-values (perm p), and if significant (perm p ≦0.05), permutation ANOVA (perm ANOVA) with Tukey post hoc (estimates (M), standard error (SE), t-value (t) and p-value (p)) according to the HD Integrated Staging System (ISS). Non-applicable (na)*

| **Proportion of** | **t test** | **mean of HC** | **mean of HD** | **p** | **perm p** | **perm ANOVA** | **perm p** | **tukey** |
| --- | --- | --- | --- | --- | --- | --- | --- | --- |
| 0 | -3.59 (64.22) | 0.203 | 0.313 | ≦0.001 | ≦0.001 | F(3,133)=5.57 | ≦0.001 | HD ISS 2 - HC:M=0.11812, SE=0.03851,t=3.067,p=0.01378  HD ISS 3 - HC:M=0.11941, SE=0.03192, t=3.741, p=0.00157 |
| 1 | 2.09 (71.56) | 0.530 | 0.472 | 0.040 | 0.045 | F(3,133)=1.60 | 0.270 | No significant pairwise group difference |
| 2 | 1.48 (59.52) | 0.238 | 0.196 | 0.144 | 0.126 | na | na | na |
| 3 | 1.19 (47.06) | 0.030 | 0.017 | 0.240 | 0.171 | na | na | na |

**Table 6. Multilinear regressions after a stepwise bidirectional approach for the depression PBA sub-scale**

*Huntington’s Disease (HD) gene carriers are grouped according to the Integrating staging system (ISS). Factors’ significance is displayed by p-values ≤ 0.05. The table shows the predictors, estimates, confidence interval (CI), and p-values (p) of the final stepwise bidirectional multiple linear regression.*

|  | **Depression sub-scale** | | |
| --- | --- | --- | --- |
| ***Predictors*** | ***Estimates*** | ***CI*** | ***p*** |
| (Intercept) | 25.40 | 4.44 – 46.36 | **0.018** |
| HD ISS 0-1 | 1.61 | -1.08 – 4.29 | 0.241 |
| HD ISS 2 | 0.66 | -2.24 – 3.55 | 0.656 |
| HD ISS 3 | 3.49 | 1.14 – 5.85 | **0.004** |
| Nervous | 117.40 | 28.53 – 206.28 | **0.010** |
| Anxious | 121.18 | 46.63 – 195.73 | **0.001** |
| Apathetic | 16.87 | -1.41 – 35.15 | 0.070 |
| Depressed | 43.18 | -7.70 – 94.06 | 0.096 |
| Disoriented | -62.02 | -117.72 – -6.32 | **0.029** |
| Pessimistic | 32.15 | -9.82 – 74.11 | 0.133 |
| Stressed | -114.85 | -252.05 – 22.36 | 0.101 |
| Activation = 0 | -24.29 | -46.55 – -2.02 | **0.033** |
| Activation = 1 | -24.38 | -45.10 – -3.66 | **0.021** |
| Activation = 2 | -22.63 | -46.98 – 1.73 | 0.069 |
| Observations | 131 | | |
| R^2^ | 0.317 | | |

**Table 7. Multilinear regressions after a stepwise bidirectional approach for the irritability/aggressivity PBA sub-scale**

*Huntington’s Disease (HD) gene carriers are grouped according to the Integrating staging system (ISS). Factors’ significance is displayed by p-values ≤ 0.05. The table shows the predictors, estimates, confidence interval (CI), and p-values (p) of the final stepwise bidirectional multiple linear regression.*

|  | **Irritability/Aggressivity sub-scale** | | |
| --- | --- | --- | --- |
| ***Predictors*** | ***Estimates*** | ***CI*** | ***p*** |
| (Intercept) | 6.40 | -1.68 – 14.47 | 0.121 |
| Apathetic | 5.20 | -1.34 – 11.74 | 0.119 |
| Other (affective phenomena) | 3.09 | -0.61 – 6.79 | 0.102 |
| Quick tempered | 14.69 | 0.51 – 28.87 | **0.042** |
| Frustrated | 7.14 | 0.42 – 13.87 | **0.037** |
| Pessimistic | 13.98 | -0.02 – 27.97 | 0.050 |
| Activation = 0 | -10.75 | -18.49 – -3.01 | **0.007** |
| Activation = 1 | -8.42 | -15.63 – -1.22 | **0.022** |
| Activation = 2 | -9.63 | -18.06 – -1.20 | **0.025** |
| Sad (primary) | 4.84 | 0.30 – 9.39 | **0.037** |
| Neutral (primary) | 3.60 | -0.33 – 7.52 | 0.072 |
| Observations | 131 | | |
| R^2^ | 0.190 | | |

**Table 8. Multilinear regressions after a stepwise bidirectional approach for the apathy PBA sub-scale**

*Huntington’s Disease (HD) gene carriers are grouped according to the Integrating staging system (ISS). Factors’ significance is displayed by p-values ≤ 0.05. The table shows the predictors, estimates, confidence interval (CI), and p-values (p) of the final stepwise bidirectional multiple linear regression.*

|  | **Apathy sub-scale** | | |
| --- | --- | --- | --- |
| ***Predictors*** | ***Estimates*** | ***CI*** | ***p*** |
| (Intercept) | 6.87 | -1.19 – 14.92 | 0.095 |
| HD ISS 0-1 | 0.10 | -0.91 – 1.11 | 0.851 |
| HD ISS 2 | -0.56 | -1.65 – 0.54 | 0.320 |
| HD ISS 3 | 0.76 | -0.13 – 1.65 | 0.095 |
| Apathetic | 15.99 | 9.29 – 22.69 | **<0.001** |
| Confused | 5.52 | -0.56 – 11.59 | 0.075 |
| Depressed | 24.17 | 5.73 – 42.62 | **0.010** |
| Hopeless | 12.22 | 1.97 – 22.46 | **0.019** |
| Distressed | -16.85 | -41.20 – 7.51 | 0.175 |
| Pessimistic | 27.20 | 11.15 – 43.25 | **0.001** |
| Activation = 0 | -11.37 | -19.60 – -3.14 | **0.007** |
| Activation = 1 | -6.67 | -14.45 – 1.11 | 0.093 |
| Activation = 2 | -10.70 | -19.82 – -1.59 | **0.021** |
| Neutral (primary) | 3.75 | 0.17 – 7.32 | **0.040** |
| Observations | 131 | | |
| R^2^ | 0.414 | | |

**Table 9. Multilinear regressions after a stepwise bidirectional approach for the obsessive/compulsive PBA sub-scale**

*Huntington’s Disease (HD) gene carriers are grouped according to the Integrating staging system (ISS). Factors’ significance is displayed by p-values ≤ 0.05. The table shows the predictors, estimates, confidence interval (CI), and p-values (p) of the final stepwise bidirectional multiple linear regression.*

|  | **Obsessive/compulsive sub-scale** | | | |
| --- | --- | --- | --- | --- |
| ***Predictors*** | ***Estimates*** | ***CI*** |  | ***p*** |
| (Intercept) | 0.62 | -0.20 – 1.44 |  | 0.140 |
| HD ISS 0-1 | -0.01 | -1.07 – 1.05 |  | 0.985 |
| HD ISS 2 | 0.27 | -0.82 – 1.36 |  | 0.624 |
| HD ISS 3 | 2.35 | 1.45 – 3.26 |  | **<0.001** |
| Anxious | -22.93 | -48.94 – 3.09 |  | 0.084 |
| Apathetic | 12.84 | 5.58 – 20.11 |  | **0.001** |
| Depressed | 17.17 | -2.45 – 36.80 |  | 0.086 |
| Fearful | 179.90 | 126.44 – 233.37 |  | **<0.001** |
| Activation = 0 | -2.76 | -5.23 – -0.30 |  | **0.028** |
| Observations | 131 | | | |
| R^2^ | 0.512 | | | |

**Table 10. Post-hoc analysis**

*Post-hoc analysis: Pearson association between participants‘ total speech time (seconds) and demographic information (education, height, weight), cognitive scores (verbal fluency, symbol digit modalities test (SDMT), Stroop word, colour and interference). For medication, total speech of Huntington’s Disease individuals (HD) taking antidepressant, anxiolytic, antipsychotic, neurological or other medication, compared to those not taking any. This was performed with t-tests. Only 92 HD individuals had medication reported.*

| Continous variable |  |  | Pearson | p-value |
| --- | --- | --- | --- | --- |
| Education |  |  | 0.216 | 0.029600 |
| Height |  |  | 0.119 | 0.234600 |
| Weight |  |  | -0.070 | 0.613200 |
| Verbal fluency |  |  | 0.291 | 0.002954 |
| SDMT |  |  | 0.154 | 0.121700 |
| Stroop word |  |  | 0.190 | 0.057090 |
| Stroop colour |  |  | 0.117 | 0.246300 |
| Stroop interference |  |  | 0.159 | 0.113100 |
| Binary variable (N) | Total speech time (second) | |  |  |
|  | Not taking medication | Taking medication | T-statistic | p-value |
| Antidepressant (71/92) | 360 | 274 | 1.673 | 0.105400 |
| Anxiolytic  (35/92) | 306 | 273 | 0.857 | 0.394300 |
| Antipsychotic  (52/92) | 354 | 247 | 2.852 | 0.005428 |
| Neurological  (14/92) | 287 | 332 | -0.577 | 0.573200 |
| Other  (50/92) | 297 | 291 | 0.157 | 0.875500 |
